# Acute and 28-days subacute toxicity studies of Gαq-RGS2 signaling inhibitor

**DOI:** 10.1186/s42826-021-00093-1

**Published:** 2021-07-26

**Authors:** Jayesh V. Beladiya, Anita A. Mehta

**Affiliations:** Department of Pharmacology, L. M. College of Pharmacy, Navarangpura, Gujarat 380009 Ahmedabad, India

**Keywords:** Acute toxicity, Sub-acute toxicity, Gαq-RGS2 signaling inhibitor, 28-days repeated dose toxicity, Single oral dose toxicity

## Abstract

**Background:**

The aim of study was to evaluate the single oral dose and 28 day repeated oral administration toxicity profile of the synthetic compound Gαq-RGS2 signaling inhibitor, (1-(5-chloro-2-hydroxyphenyl)-3-(4-(trifluoromethyl)phenyl)-1 H-1,2,4-triazol-5(4 H)-one) as per OECD guideline 425 (2008a) and 407 (2008b), respectively.

**Results:**

In acute toxicity study, a single oral dose administration of Gαq-RGS2 signaling inhibitor did not show any mortality at doses of 5, 50, 300 and 2000 mg/kg within 24 h and 14 days. The treatment of Gαq-RGS2 signaling inhibitor at dose 10 and 100 mg/kg for 28 days did not show any mortality, significant changes in the increase of body weight, various organ damage markers, hematological parameters, relative organ/body weight ratio and microscopic anatomical texture of essential organs as compared to vehicle and normal control.

**Conclusions:**

A single oral administration of Gαq-RGS2 signaling inhibitor up to dose of 2000 mg/kg in mice and repeated administration of Gαq-RGS2 signaling inhibitor at higher dose 100 mg/kg for 28 days in the rats is safe.

**Supplementary Information:**

The online version contains supplementary material available at 10.1186/s42826-021-00093-1.

## Background

The major barrier for drug failure in the late stage of drug development process is toxicity and adverse effects. A troglitazone was withdrawn from the market due to identification of the unexpected liver toxicity as adverse effect in the later phase of drug surveillance phase [1]. Therefore, it is necessary to prior collect the safety and toxicity data of the novel chemical entity in the animals before exploring in the clinical study. It is also required to assess the organ specific toxicity profile of the novel chemical entity at preclinical level. Therefore, the aim of current study was to establish the acute and subacute toxicity profile of the trizolone ring bearing compound, Gαq-RGS2 signaling inhibitor in the early phase of drug discovery in the rodents.

The synthetic compound Gαq-RGS2 signaling inhibitor, (1-(5-chloro-2-hydroxyphenyl)-3-(4-(trifluoromethyl)phenyl)-1 H-1,2,4-triazol-5(4 H)-one) is a triazolone ring containing compound [2]. Triazolone ring bearing compounds are emerging class of therapeutic target in the drug development. Recently, novel chemical entity containing triazolone ring, Ganetespib has been identified and extensively studied the anticancer activity of Ganetespib in the animals. In which Ganetespib showed the anticancer activity in both *in-vitro* and *in-vivo* preclinical studies [3]. Previous reported study synthesized the triazolone bearing compound Gαq-RGS2 signaling inhibitor and extensively evaluated the pharmacological activity against the urinary incontinence, which showed the beneficial activity in the urinary incontinence [4]. Furthermore, the Gαq-RGS2 signaling inhibitor has demonstrated Gαq signaling inhibitor activity by acting at the intersection of RGS2 and G-αq proteins, resulting in attenuated the Gαq signaling which reduced calcium fluxes and reduced muscle contraction [5]. In our previous study, we showed the beneficial effect of Gαq-RGS2 signaling inhibitor in the aminophylline induced cardiac arrhythmia [6]. It has been documented that the RGS2 deficient mice are hypertensive in nature because of renovascular abnormalities [7]. An increased contractile response to angiotensin II was observed in the isolated mesenteric arteries from RGS2-/- mice [8]. Thus, RGS2 is potent therapeutic target for cardiovascular diseases and future progress in the development of this class of drugs and Gαq-RGS2 signaling inhibitors for treatment of cardiovascular diseases is crucial and beneficial.

## Results

### Acute toxicity

A single administration of Gαq-RGS2 signaling inhibitor did not show the any mortality up to dose of 2000 mg/kg within 24 h and 14 days. None of the animals showed the alteration in the muscle activity, reflex activity and secretory during the observation period (14 days) after administration of Gαq-RGS2 signaling inhibitor (Supplemental data available).

### Subacute toxicity

**Fig. 1:**
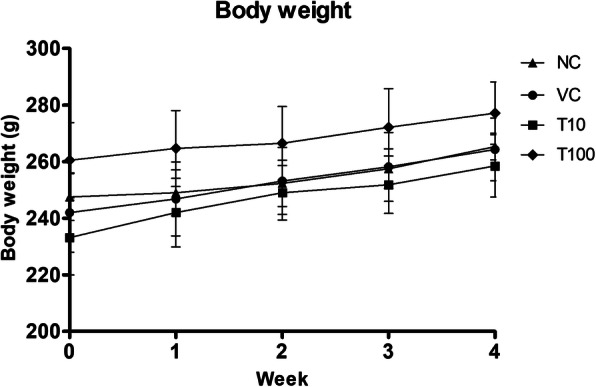
Effect of Gαq-RGS2 signaling inhibitor on body weight (*n* = 6) NC = Normal control, VC = 1 % DMSO treated normal rats, T10 = Gαq-RGS2 signaling inhibitor (10 mg/kg) treated rats and T100 = Gαq-RGS2 signaling inhibitor (100 mg/kg) treated rats

The repeated administration of vehicle (1 % v/v DMSO) and Gαq-RGS2 signaling inhibitor at doses of 10 and 100 mg/kg/d for 28 days did not show the any mortality and clinical abnormalities as compared to normal control. The increase in the body weight of rats treated with vehicle and Gαq-RGS2 signaling inhibitor did not show the significant difference as compared to normal control at weekly interval on 7th, 14th, 21st and 28th day (Fig. 1).

**Fig. 2:**
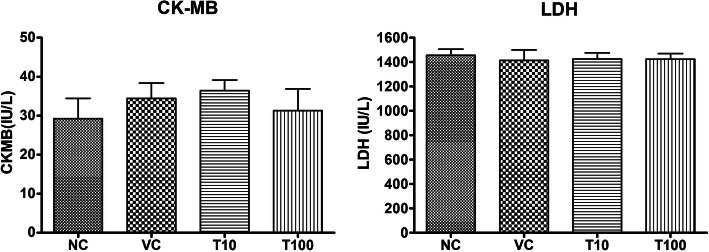
Effect of Gαq-RGS2 signaling inhibitor on cardiac damage markers (*n* = 6) NC = Normal control, VC = 1 % DMSO treated normal rats, T10 = Gαq-RGS2 signaling inhibitor (10 mg/kg) treated rats and T100 = Gαq-RGS2 signaling inhibitor (100 mg/kg) treated rats

**Fig. 3:**
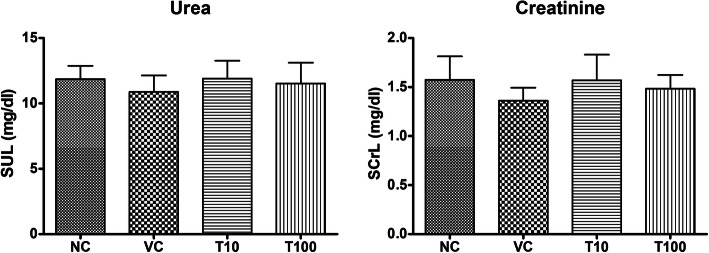
Effect of Gαq-RGS2 signaling inhibitor on kidney damage markers (*n* = 6) NC = Normal control, VC = 1 % DMSO treated normal rats, T10 = Gαq-RGS2 signaling inhibitor (10 mg/kg) treated rats and T100 = Gαq-RGS2 signaling inhibitor (100 mg/kg) treated rats

**Fig. 4:**
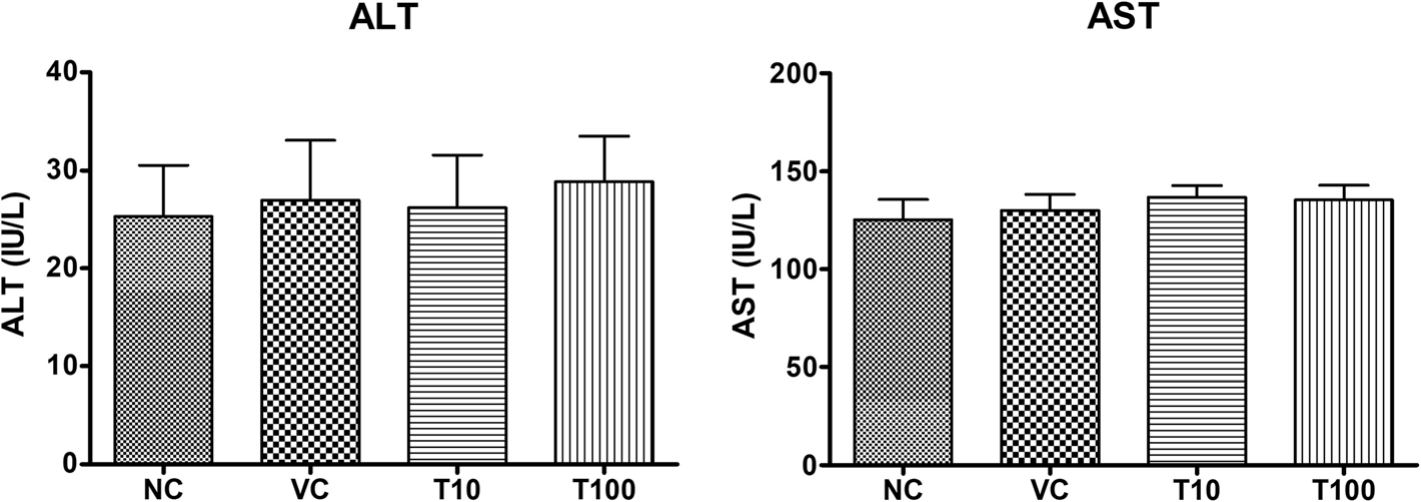
Effect of Gαq-RGS2 signaling inhibitor on liver damage markers (*n* = 6) NC = Normal control, VC = 1 % DMSO treated normal rats, T10 = Gαq-RGS2 signaling inhibitor (10 mg/kg) treated rats and T100 = Gαq-RGS2 signaling inhibitor (100 mg/kg) treated rats

**Fig. 5:**
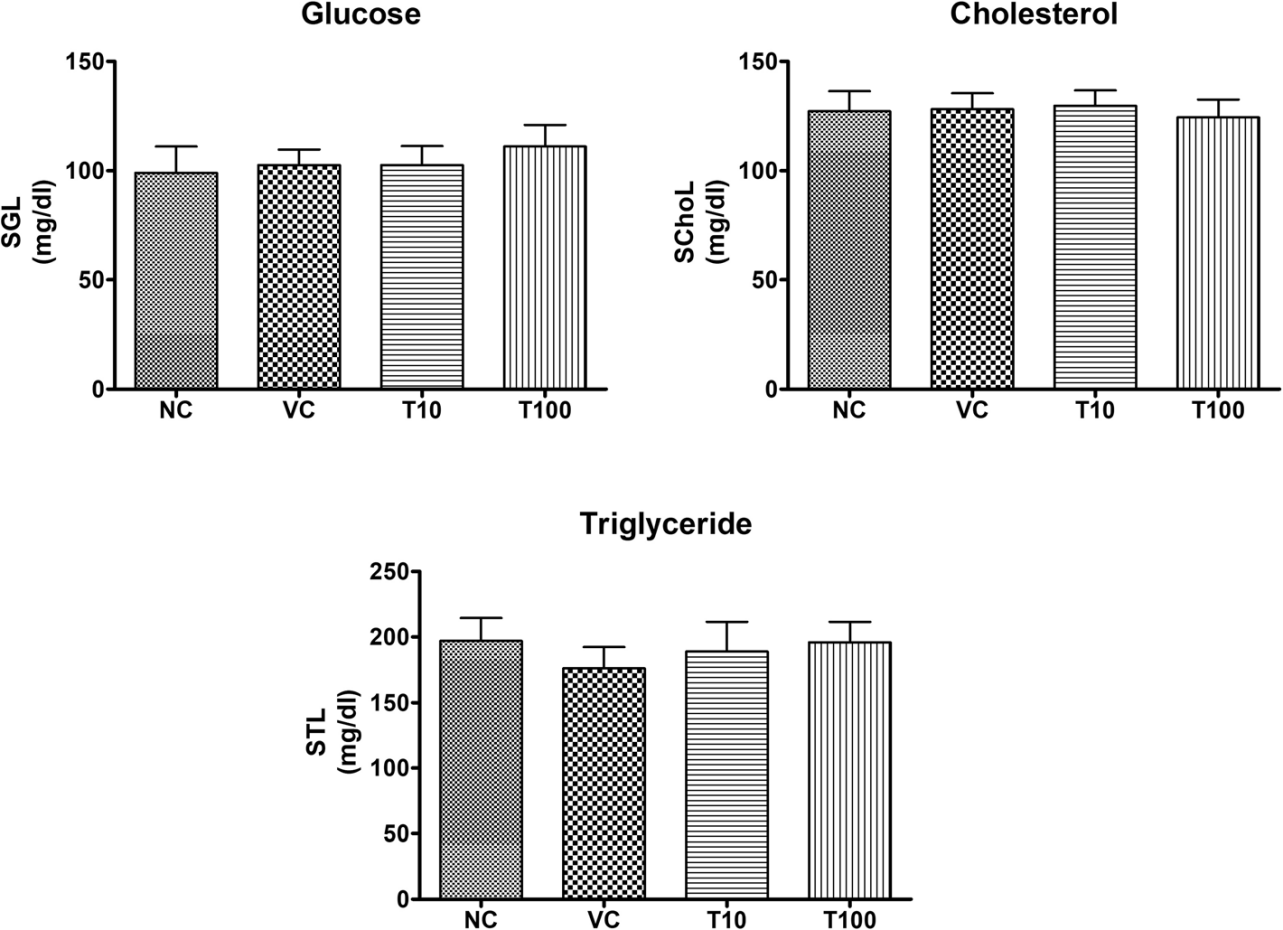
Effect of Gαq-RGS2 signaling inhibitor on metabolic parameters (*n* = 6) NC = Normal control, VC = 1 % DMSO treated normal rats, T10 = Gαq-RGS2 signaling inhibitor (10 mg/kg) treated rats and T100 = Gαq-RGS2 signaling inhibitor (100 mg/kg) treated rats

**Fig. 6:**
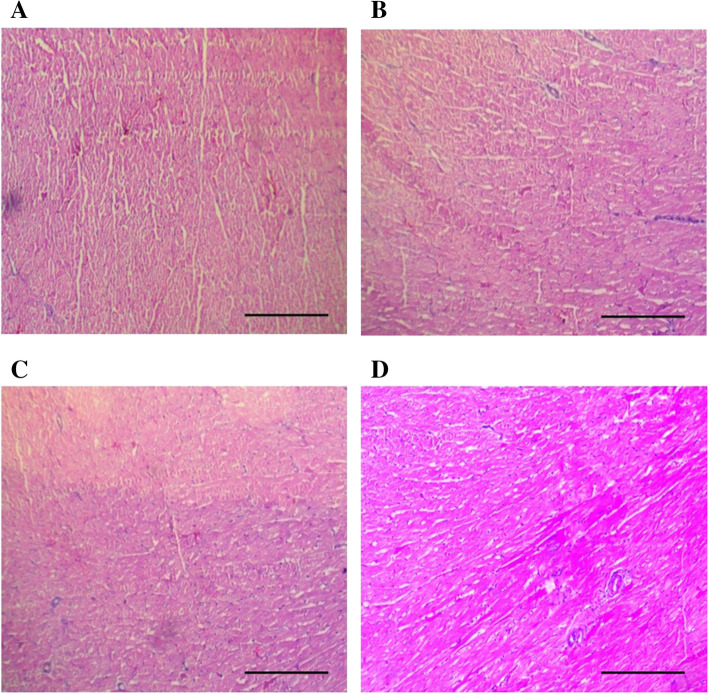
Photomicrographs of rat heart stained with haematoxylin and eosin (Scale bar = 100 μm, original magnification x 100). **A** = Normal control (NC), **B** = 1 % DMSO treated normal rats (VC), **C** = Gαq-RGS2 signaling inhibitor (10 mg/kg) treated normal rats (T10) and **D** = Gαq-RGS2 signaling inhibitor (100 mg/kg) treated normal rats (T100)

**Fig. 7:**
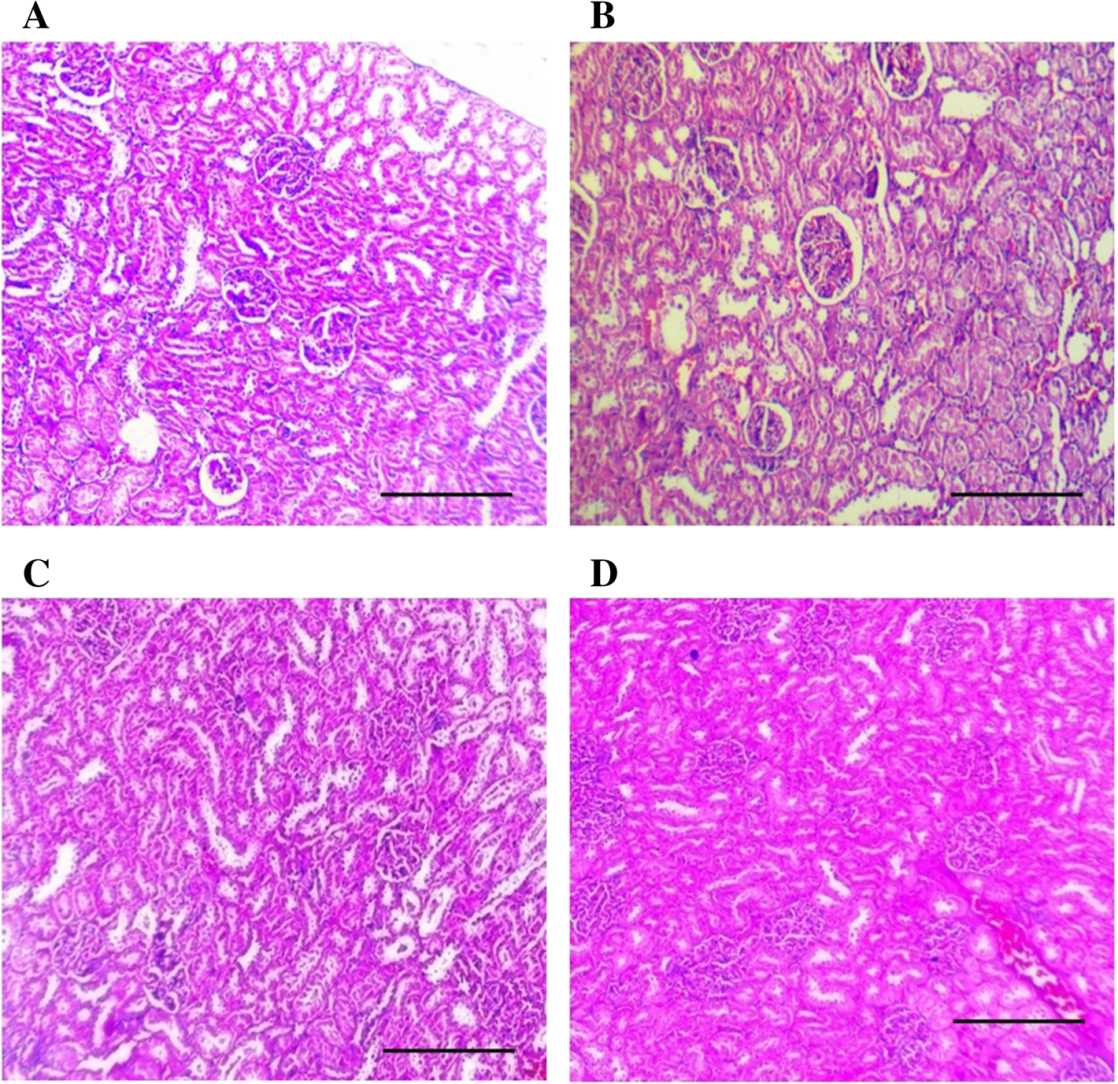
Photomicrographs of rat kidney stained with haematoxylin and eosin (Scale bar = 100 μm, original magnification x 100). **A** = Normal control (NC), **B** = 1 % DMSO treated normal rats (VC), **C** = Gαq-RGS2 signaling inhibitor (10 mg/kg) treated rats (T10) and **D** = Gαq-RGS2 signaling inhibitor (100 mg/kg) treated rats (T100)

**Fig. 8:**
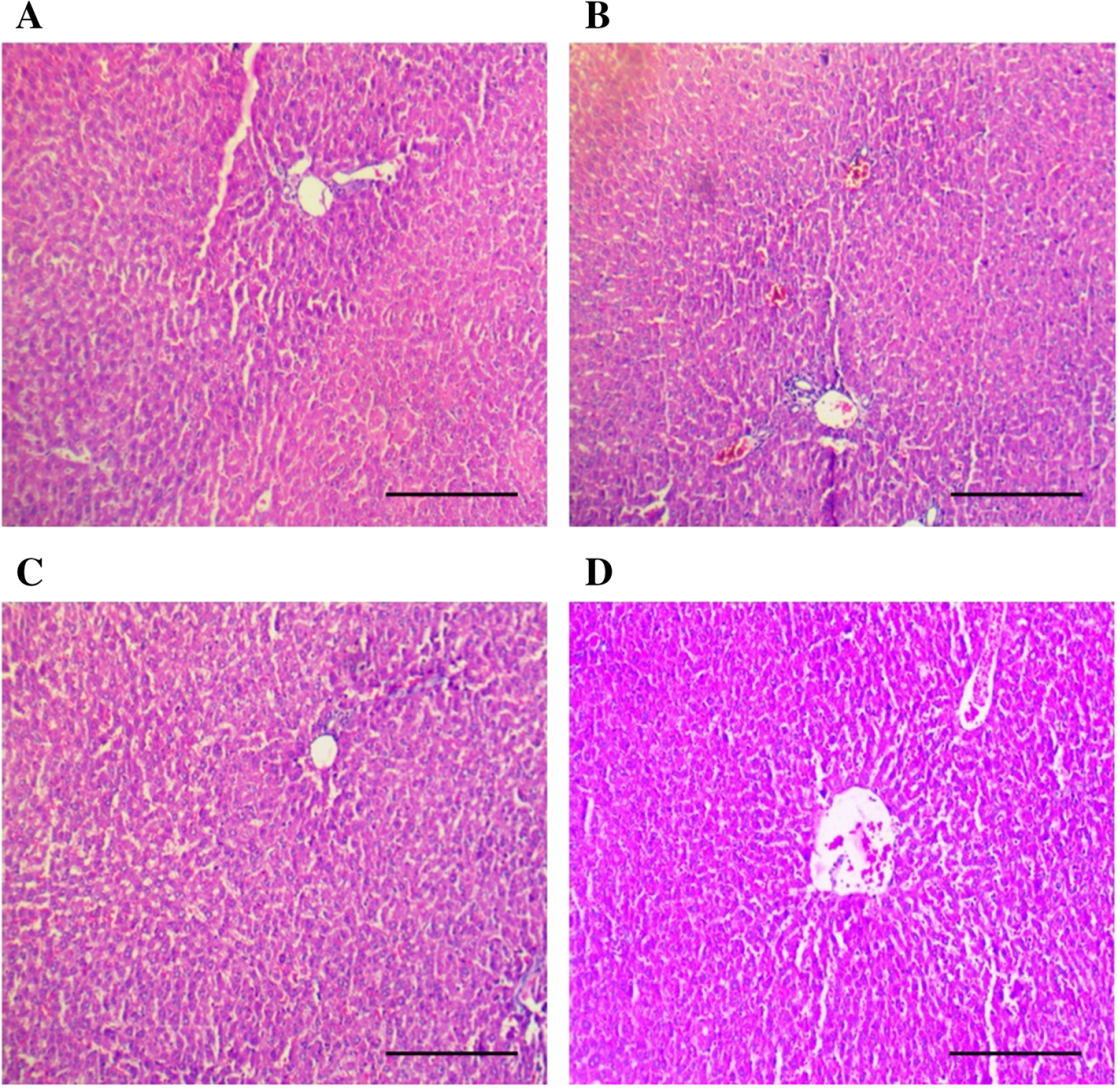
Photomicrographs of rat liver stained with haematoxylin and eosin (Scale bar = 100 μm, original magnification x 100). **A** = Normal control (NC), **B** = 1 % DMSO treated normal rats (VC), **C** = Gαq-RGS2 signaling inhibitor (10 mg/kg) treated rats (T10) and **D** = Gαq-RGS2 signaling inhibitor (100 mg/kg) treated rats (T100)

**Fig. 9:**
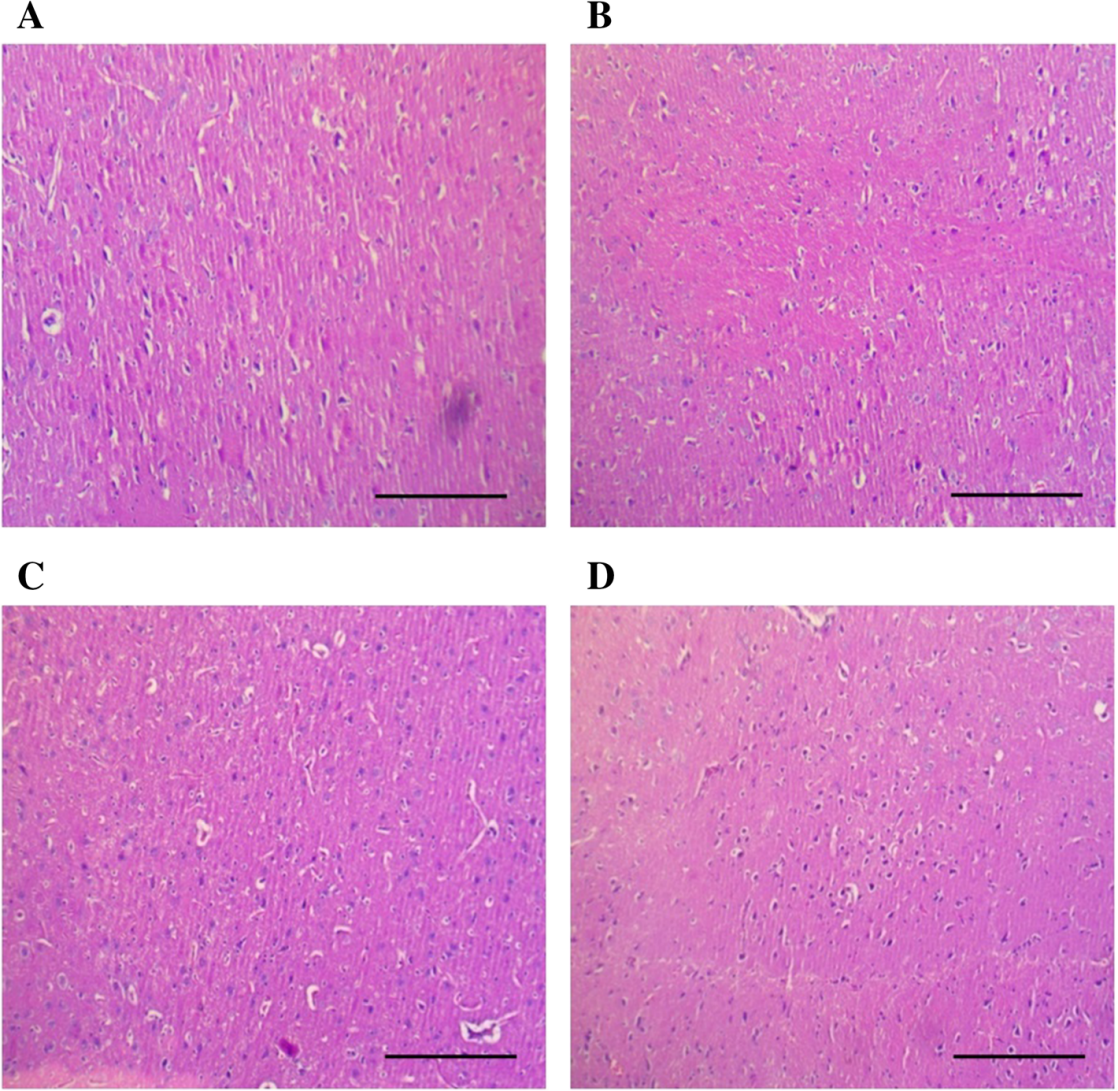
Photomicrographs of rat brain stained with haematoxylin and eosin (Scale bar = 100 μm, original magnification x 100). **A** = Normal control (NC), **B** = 1 % DMSO treated normal rats (VC), **C** = Gαq-RGS2 signaling inhibitor (10 mg/kg) treated normal rats (T10) and **D** = Gαq-RGS2 signaling inhibitor (100 mg/kg) treated normal rats (T100)

**Fig. 10:**
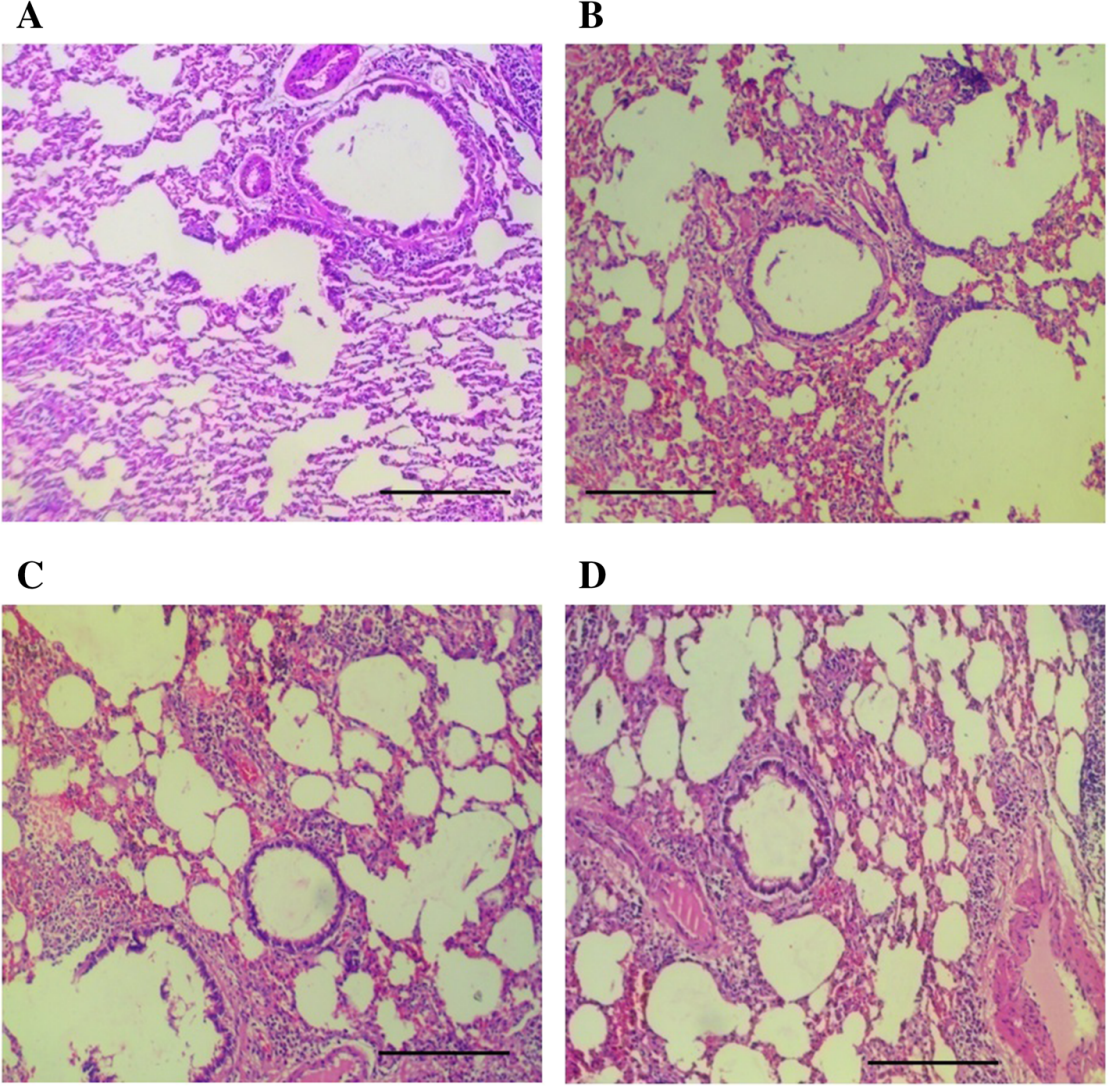
Photomicrographs of rat lung stained with haematoxylin and eosin (Scale bar = 100 μm, original magnification x 100). **A** = Normal control (NC), **B** = 1 % DMSO treated normal rats (VC), **C** = Gαq-RGS2 signaling inhibitor (10 mg/kg) treated rats (T10) and **D** = Gαq-RGS2 signaling inhibitor (100 mg/kg) treated rats (T100)

The treatment of vehicle (1 % v/v DMSO) and Gαq-RGS2 signaling inhibitor at doses of 10 and 100 mg/kg/d for 28 days did not show significant changes in the hematological parameters such as Red blood cells (RBC) count, total and differential white blood cells (WBC) count and hemoglobin (Hb) content as compared to normal control (Table 1). Various organ damage markers such as cardiac damage (Creatine Kinase (CK-MB), Lactate dehydrogenase (LDH)) (Fig. 2), kidney damage (urea, creatinine) (Fig. 3), liver damage markers (Alanine Aminotransferase (ALT), Aspartate aminotransferase (AST)) (Fig. 4) and metabolic parameters (glucose, cholesterol, and triglyceride) (Fig. 5) did not significantly alter by treatment of vehicle (1 % v/v DMSO) and Gαq-RGS2 signaling inhibitor as compared to normal control. Furthermore, vehicle and Gαq-RGS2 signaling inhibitor treated rats did not show the significant difference in the absolute and relative weights of the essential organs as compared to normal control (Table 2). In the histological examination, various damages in the organs such as neutrophil infiltration and hemorrhage in the heart, glomerular atrophy and necrotic convoluted tubules in the kidney, fat accumulation and necrosis in the liver, alveoli obstruction and neutrophil infiltration in lung were examined. The histological examination of essential organs did not show the significant damage in the heart (Fig. 6), kidney (Fig. 7), liver (Fig. 8), brain (Fig. 9) and lung (Fig. 10) as compared to normal control. These results revealed that an oral administration of Gαq-RGS2 signaling inhibitor at doses of 10 and 100 mg/kg for 28 days in rats did not produce significant toxic or adverse effects. Furthermore, there were no significant changes observed in the hematological and biochemical parameters between genders as compared to normal control.

**Table 1:** Effect of Gαq-RGS2 signaling inhibitor on hematological parameters

Parameters	NC	VC	T10	T100
RBC (x10^6^ cells/mm^3^)	3.745 ± 0.556	3.945 ± 0.556	3.712 ± 0.779	3.583 ± 0.774
Total WBC (x10^3^ cells/mm^3^)	6.533 ± 1.244	5.833 ± 0.836	6.317 ± 1.036	6.583 ± 1.049
Granulocytes (x10^3^ cells/mm^3^)	4.885 ± 1.003	5.234 ± 0.668	4.693 ± 0.831	5.53 ± 0.688
Lymphocytes (x10^3^ cells/mm^3^)	2.033 ± 0.274	2.233 ± 0.287	2.417 ± 0.217	2.383 ± 0.277
Monocytes (x10^3^ cells/mm^3^)	0.050 ± 0.012	0.057 ± 0.012	0.060 ± 0.011	0.058 ± 0.012
Platelets (x10^3^ cells/µL^3^)	265.4 ± 39.30	257.4 ± 38.32	248.8 ± 29.10	257.8 ± 42.12
Hb content (g%)	13.48 ± 1.32	13.20 ± 1.07	13.92 ± 1.60	13.35 ± 0.84

NC = Normal control, VC = 1 % DMSO treated normal rats, T10 = Gαq-RGS2 signaling inhibitor (10 mg/kg) treated rats and T100 = Gαq-RGS2 signaling inhibitor (100 mg/kg) treated rats. (*n* = 6/each group)

**Table 2:** Effect of Gαq-RGS2 signaling inhibitor on organ-to body weight ratio (%) of various organs

Organ	NC	VC	T10	T100
Heart	0.289 ± 0.026	0.268 ± 0.015	0.290 ± 0.019	0.284 ± 0.027
Kidney	0.544 ± 0.04	0.534 ± 0.075	0.608 ± 0.07	0.562 ± 0.063
Liver	2.010 ± 0.222	1.929 ± 0.207	2.263 ± 0.221	2.175 ± 0.287
Brain	0.681 ± 0.018	0.690 ± 0.035	0.752 ± 0.032	0.659 ± 0.019
Lung	0.331 ± 0.036	0.346 ± 0.029	0.393 ± 0.036	0.387 ± 0.011

NC = , Normal control, VC = 1 % DMSO treated normal rats, T10 = Gαq-RGS2 signaling inhibitor (10 mg/kg) treated rats and T100 = Gαq-RGS2 signaling inhibitor (100 mg/kg) treated rats. (*n* = 6/each group)

## Discussion

The current study showed the acute and subacute toxicity profile of the Gαq-RGS2 signaling inhibitor in the rodents. In acute oral dose toxicity study, a single oral administration of Gαq-RGS2 signaling inhibitor at the dose up to 2000 mg/kg in the female mice did not show the mortality and adverse symptoms. As per Hodge and Sterner Scale, Gαq-RGS2 signaling inhibitor is fall under the slightly toxic category [9].

The OECD guideline 407 was followed for 28 days repeated oral dose toxicity study in the rats. The two doses 10 and 100 mg/kg of Gαq-RGS2 signaling inhibitor were used in the subacute toxicity study. These doses in subacute toxicity were selected from the effective dose (10 mg/kg) of Gαq-RGS2 signaling inhibitor against the cardiac arrhythmia in the rats in our previous study [6]. Therefore, we have evaluated the subtoxicity profile of Gαq-RGS2 signaling inhibitor at same dose (10 mg/kg) and ten times higher dose (100 mg/kg). In 28-days repeated oral dose toxicity study, there was no mortality, no adverse effects or toxic effects observed during or end of the study. The treatment of Gαq-RGS2 signaling inhibitor did not show the significant modification in the rising of body weight between the rats of all the groups. Various biochemical parameters such as cardiac damage markers, liver damage markers, kidney damage markers and metabolic parameters did not alter by the treatment of Gαq-RGS2 signaling inhibitor. The alteration in the specific organ damage marker indicates the beginning of reversible or irreversible injury of the organ. The liver is more prone to the adverse effects as compared to other organs because it involves in the biotransformation and excretion process. A long time exposure to chemical entity might be cause the imbalance of oxidative-antioxidant system in the hepatocytes and liver dysfunctions [10–13]. In the current study, percent organ to body weight ratio and anatomical texture of essential organs (liver, heart, kidney, lungs and brain) were evaluated. The previous reported studies also suggested the percent organ to body weights ratio as susceptible indicator to evaluate the toxic effects of drugs [14]. The exposure of Gαq-RGS2 signaling inhibitor did not show the significant alteration in the percent organ to body weight ratio and anatomical texture of heart, kidney, lungs, liver and brain.

## Conclusions

The Gαq-RGS2 signaling inhibitor did not produce any mortality on single oral administration up to dose of 2000 mg/kg in the mice. The treatment of Gαq-RGS2 signaling inhibitor at doses of 10 and 100 mg/kg in the rats for 28 days did not produce any lethality and abnormalities on metabolic and hematological parameters, various organ damage markers and essential organs.

## Methods

### Animals

A permission to conduct the experiment (Protocol number: LMCP/COLOGY/15/13; Date: 22/12/2015) was taken from Institutional Animal Ethics Committee (IAEC). A guideline of Committee for the Purpose of Control and Supervision of Experiments on Animals (CPCSEA) was followed throughout the experiment. Albino mice (20–25 g) and Sprague-Dawley rats (200–250 g) were obtained at 1 week prior the starting of study from Zydus research center (ZRC), Ahmedabad, India. Animals were free access to standard pellet diet and filtered tap water. They were maintained at 22 ± 1 °C, 55 ± 5 % relative humidity and 12-hr light-dark cycle in the animal house facility of institute.

### Chemicals

Gαq-RGS2 signaling inhibitor (1-(5-chloro-2-hydroxyphenyl)-3-(4-(trifluoromethyl)phenyl)-1 H-1,2,4-triazol-5(4 H)-one) was synthesized and purified in our laboratory according to reported data [2].

### Acute toxicity study

A single oral administration of Gαq-RGS2 signaling inhibitor was performed in the albino mice as per the OECD guideline 425 (OECD, 2008a). The Gαq-RGS2 signaling inhibitor was dissolved in the absolute DMSO and desired concentration of drug and DMSO (1 % v/v) was achieved with distilled water. In the control group, mice were treated with vehicle (DMSO, 1 % v/v). The Gαq-RGS2 signaling inhibitor was administered subsequentially in the ascending order of dose 5, 50, 300 and 2000 mg/kg in the respective group. The Gαq-RGS2 signaling inhibitor was administered in the next group after 48 h survival of animals. The mice were fasted overnight (drinking water *ad libitum*) prior to dosing and food was restricted further 4 h after dosing. The mortality and clinical observations such as muscle activity (Locomotion, muscle co-ordination, catatonia and convulsive episode), reflex activity (Visual place response, whriting response, Tail pinch response, piloerection) and secretory activity (Lacrimation, salivation, sniffing and defecation) were observed at 1 h, 2 h, 4 h, and 24 h after dosing and subsequentialy once daily for 14 days [15, 16].

### Subacute toxicity study

The subacute toxicity was performed as per OECD guideline 407 for Testing of Chemical: Repeated Dose 28-days Oral Toxicity Study in Rodents (OECD, 2008b). The rats were randomly divided into four groups; normal control, vehicle control and two treatment groups (10 and 100 mg/kg treated). Normal control group was treated with saline (10 ml/kg/d, 28 days). Vehicle control group was treated with DMSO (1 % v/v, 10ml/kg/d, 28 days). The Gαq-RGS2 signaling inhibitor was administered at the doses of 10 and 100 mg/kg/d, for 28 days in the individual treatment group, respectively. These two doses were selected on basis of our All the animals were examined once daily for clinical signs, mortality and morbidity throughout the study. Body weights of rats were measured on day 1 before dosing followed by once a week during study. At the end of study, various organ damage markers, hematological parameters, relative organ/body weight ratio and histological examination of essential organs were performed [17].

### Biochemical parameters

Blood was collected from retro-orbital plexus of anesthetized rats. It was kept at room temperature for 30 min, centrifuged at 1000 g for 15 min, and serum was isolated by aspiration. Various biochemical parameters such as liver damage markers (ALT, AST) and metabolic abnormalities (glucose, total cholesterol and triglyceride), cardiac damage markers (CK-MB, LDH), kidney damage markers (urea, creatinine) were determined using spectrophotometer based kits (Span Diagnostic Ltd., Surat, Gujarat, India) [18].

### Hematological methods

The blood was collected through heart puncture under ketamine (100 mg/ml)/xylazine (20 mg/ml) anesthesia in the EDTA coated tubes. The hematological parameters including hemoglobin concentration, erythrocytes, total and differential WBC (Granulocytes, lymphocytes, monocytes) and thrombocytes count were measured in the collected blood using Auto cell analyzer (Dia-count 60, Diatek, India) [19].

### Relative organ/body weight and histological examination

Rats were sacrificed and essential organs heart, liver, kidney, brain and lungs were isolated. A weight of each organ was measured using weighing balance. An organ-to-final-body-weight ratio was calculated for each organ [20]. All the isolated organs were washed with saline and fixed in the 10 % neutral buffered formalin. All the specimens were sent for section and staining with hematoxylin and eosin dye. Images of selected sections were captured at original (100x) magnifications using a zoom digital camera (MLX, Magnus, China) [19, 21].

### Statistical analysis

Data were expressed as Mean ± SEM. Statistical analysis was performed by one way analysis of variance (ANOVA), followed by Bonferoni’s multiple comparison test using Graph pad prism 5.0 software. *P* < 0.05 was considered as statistically significant.

## Supplementary Information

Additional file 1: **Table 1. **Effect of single oral dose treatment of Gαq-RGS2 signaling inhibitor on mortality. **Table 2.**Effect of single oral dose treatment of Gαq-RGS2 signaling inhibitor on locomotion. **Table 3. **Effect of single oral dose treatment of Gαq-RGS2 signaling inhibitor on muscle co-ordination. **Table 4. **Effect of single oral dose treatment of Gαq-RGS2 signaling inhibitor on catatonia. **Table 5. **Effect of single oral dose treatment of Gαq-RGS2 signaling inhibitor on convulsive episode. **Table 6. **Effect of single oral dose treatment of Gαq-RGS2 signaling inhibitor on visual place response. **Table 7. **Effect of single oral dose treatment of Gαq-RGS2 signaling inhibitor on writhing response. **Table 8. **Effect of single oral dose treatment of Gαq-RGS2 signaling inhibitor on tail pinch response. **Table 9. **Effect of single oral dose treatment of Gαq-RGS2 signaling inhibitor on piloerection. **Table 10. **Effect of single oral dose treatment of Gαq-RGS2 signaling inhibitor on lacrimation. **Table 11. **Effect of single oral dose treatment of Gαq-RGS2 signaling inhibitor on salivation. **Table 12. **Effect of single oral dose treatment of Gαq-RGS2 signaling inhibitor on sniffing. **Table 13. **Effect of single oral dose treatment of Gαq-RGS2 signaling inhibitor on defecation.

## Data Availability

The datasets during and/or analyzed during the current study available from the corresponding author on reasonable request.
