# Supplementary material for: Acute and 28-days subacute toxicity studies of Gαq-RGS2 signaling inhibitor
Source: Lab Anim Res. 2021 Jul 26;37:17. doi: 10.1186/s42826-021-00093-1 (PMC8314442; doi:10.1186/s42826-021-00093-1)
Supplement: Supplementary file 1 — Table 1. Effect of single oral dose treatment of Gαq-RGS2 signaling inhibitor on mortality. Table 2.Effect of single oral dose treatment of Gαq-RGS2 signaling inhibitor on locomotion. Table 3. Effect of single oral dose treatment of Gαq-RGS2 signaling inhibitor on muscle co-ordination. Table 4. Effect of single oral dose treatment of Gαq-RGS2 signaling inhibitor on catatonia. Table 5. Effect of single oral dose treatment of Gαq-RGS2 signaling inhibitor on convulsive episode. Table 6. Effect of single oral dose treatment of Gαq-RGS2 signaling inhibitor on visual place response. Table 7. Effect of single oral dose treatment of Gαq-RGS2 signaling inhibitor on writhing response. Table 8. Effect of single oral dose treatment of Gαq-RGS2 signaling inhibitor on tail pinch response. Table 9. Effect of single oral dose treatment of Gαq-RGS2 signaling inhibitor on piloerection. Table 10. Effect of single oral dose treatment of Gαq-RGS2 signaling inhibitor on lacrimation. Table 11. Effect of single oral dose treatment of Gαq-RGS2 signaling inhibitor on salivation. Table 12. Effect of single oral dose treatment of Gαq-RGS2 signaling inhibitor on sniffing. Table 13. Effect of single oral dose treatment of Gαq-RGS2 signaling inhibitor on defecation. [file 42826_2021_93_MOESM1_ESM.docx]

**Table 1.** Effect of single oral dose treatment of Gαq-RGS2 signaling inhibitor on mortality

| **Sr. No.** | **Time** | **Groups**  (Mortality/Total No. of animals) | | | | |
| --- | --- | --- | --- | --- | --- | --- |
|  |  | **NC** | **T1** | **T2** | **T3** | **T4** |
| 1 | 1h | 0/3 | 0/3 | 0/3 | 0/3 | 0/3 |
|  | 2 h | 0/3 | 0/3 | 0/3 | 0/3 | 0/3 |
|  | 4 h | 0/3 | 0/3 | 0/3 | 0/3 | 0/3 |
|  | 14 h | 0/3 | 0/3 | 0/3 | 0/3 | 0/3 |
|  | 24 h | 0/3 | 0/3 | 0/3 | 0/3 | 0/3 |
| 2 | 2 d | 0/3 | 0/3 | 0/3 | 0/3 | 0/3 |
| 3 | 3 d | 0/3 | 0/3 | 0/3 | 0/3 | 0/3 |
| 4 | 4 d | 0/3 | 0/3 | 0/3 | 0/3 | 0/3 |
| 5 | 5 d | 0/3 | 0/3 | 0/3 | 0/3 | 0/3 |
| 6 | 6 d | 0/3 | 0/3 | 0/3 | 0/3 | 0/3 |
| 7 | 7 d | 0/3 | 0/3 | 0/3 | 0/3 | 0/3 |
| 8 | 8 d | 0/3 | 0/3 | 0/3 | 0/3 | 0/3 |
| 9 | 9 d | 0/3 | 0/3 | 0/3 | 0/3 | 0/3 |
| 10 | 10 d | 0/3 | 0/3 | 0/3 | 0/3 | 0/3 |
| 11 | 11 d | 0/3 | 0/3 | 0/3 | 0/3 | 0/3 |
| 12 | 12 d | 0/3 | 0/3 | 0/3 | 0/3 | 0/3 |
| 13 | 13 d | 0/3 | 0/3 | 0/3 | 0/3 | 0/3 |
| 14 | 14 d | 0/3 | 0/3 | 0/3 | 0/3 | 0/3 |

NC = Normal control, VC = 1% DMSO treated normal rats (Vehicle control), T10 = Gαq-RGS2 signaling inhibitor (10 mg/kg) treated rats and T100 = Gαq-RGS2 signaling inhibitor (100 mg/kg) treated rats.

**Table 2.** Effect of single oral dose treatment of Gαq-RGS2 signaling inhibitor on locomotion.

| **Sr. No.** | **Time** | **Groups**  (Alteration in locomotion/Total No. of animals) | | | | |
| --- | --- | --- | --- | --- | --- | --- |
|  |  | **NC** | **T1** | **T2** | **T3** | **T4** |
| 1 | 1h | 0/3 | 0/3 | 0/3 | 0/3 | 0/3 |
|  | 2 h | 0/3 | 0/3 | 0/3 | 0/3 | 0/3 |
|  | 4 h | 0/3 | 0/3 | 0/3 | 0/3 | 0/3 |
|  | 14 h | 0/3 | 0/3 | 0/3 | 0/3 | 0/3 |
|  | 24 h | 0/3 | 0/3 | 0/3 | 0/3 | 0/3 |
| 2 | 2 d | 0/3 | 0/3 | 0/3 | 0/3 | 0/3 |
| 3 | 3 d | 0/3 | 0/3 | 0/3 | 0/3 | 0/3 |
| 4 | 4 d | 0/3 | 0/3 | 0/3 | 0/3 | 0/3 |
| 5 | 5 d | 0/3 | 0/3 | 0/3 | 0/3 | 0/3 |
| 6 | 6 d | 0/3 | 0/3 | 0/3 | 0/3 | 0/3 |
| 7 | 7 d | 0/3 | 0/3 | 0/3 | 0/3 | 0/3 |
| 8 | 8 d | 0/3 | 0/3 | 0/3 | 0/3 | 0/3 |
| 9 | 9 d | 0/3 | 0/3 | 0/3 | 0/3 | 0/3 |
| 10 | 10 d | 0/3 | 0/3 | 0/3 | 0/3 | 0/3 |
| 11 | 11 d | 0/3 | 0/3 | 0/3 | 0/3 | 0/3 |
| 12 | 12 d | 0/3 | 0/3 | 0/3 | 0/3 | 0/3 |
| 13 | 13 d | 0/3 | 0/3 | 0/3 | 0/3 | 0/3 |
| 14 | 14 d | 0/3 | 0/3 | 0/3 | 0/3 | 0/3 |

NC = Normal control, VC = 1% DMSO treated normal rats (Vehicle control), T10 = Gαq-RGS2 signaling inhibitor (10 mg/kg) treated rats and T100 = Gαq-RGS2 signaling inhibitor (100 mg/kg) treated rats.

**Table 3.** Effect of single oral dose treatment of Gαq-RGS2 signaling inhibitor on muscle co-ordination.

| **Sr. No.** | **Time** | **Groups**  (Alteration in muscle co-ordination/Total No. of animals) | | | | |
| --- | --- | --- | --- | --- | --- | --- |
|  |  | **NC** | **T1** | **T2** | **T3** | **T4** |
| 1 | 1h | 0/3 | 0/3 | 0/3 | 0/3 | 0/3 |
|  | 2 h | 0/3 | 0/3 | 0/3 | 0/3 | 0/3 |
|  | 4 h | 0/3 | 0/3 | 0/3 | 0/3 | 0/3 |
|  | 14 h | 0/3 | 0/3 | 0/3 | 0/3 | 0/3 |
|  | 24 h | 0/3 | 0/3 | 0/3 | 0/3 | 0/3 |
| 2 | 2 d | 0/3 | 0/3 | 0/3 | 0/3 | 0/3 |
| 3 | 3 d | 0/3 | 0/3 | 0/3 | 0/3 | 0/3 |
| 4 | 4 d | 0/3 | 0/3 | 0/3 | 0/3 | 0/3 |
| 5 | 5 d | 0/3 | 0/3 | 0/3 | 0/3 | 0/3 |
| 6 | 6 d | 0/3 | 0/3 | 0/3 | 0/3 | 0/3 |
| 7 | 7 d | 0/3 | 0/3 | 0/3 | 0/3 | 0/3 |
| 8 | 8 d | 0/3 | 0/3 | 0/3 | 0/3 | 0/3 |
| 9 | 9 d | 0/3 | 0/3 | 0/3 | 0/3 | 0/3 |
| 10 | 10 d | 0/3 | 0/3 | 0/3 | 0/3 | 0/3 |
| 11 | 11 d | 0/3 | 0/3 | 0/3 | 0/3 | 0/3 |
| 12 | 12 d | 0/3 | 0/3 | 0/3 | 0/3 | 0/3 |
| 13 | 13 d | 0/3 | 0/3 | 0/3 | 0/3 | 0/3 |
| 14 | 14 d | 0/3 | 0/3 | 0/3 | 0/3 | 0/3 |

NC = Normal control, VC = 1% DMSO treated normal rats (Vehicle control), T10 = Gαq-RGS2 signaling inhibitor (10 mg/kg) treated rats and T100 = Gαq-RGS2 signaling inhibitor (100 mg/kg) treated rats.

**Table 4.** Effect of single oral dose treatment of Gαq-RGS2 signaling inhibitor on catatonia.

| **Sr. No.** | **Time** | **Groups**  (Catatonia/Total No. of animals) | | | | |
| --- | --- | --- | --- | --- | --- | --- |
|  |  | **NC** | **T1** | **T2** | **T3** | **T4** |
| 1 | 1h | 0/3 | 0/3 | 0/3 | 0/3 | 0/3 |
|  | 2 h | 0/3 | 0/3 | 0/3 | 0/3 | 0/3 |
|  | 4 h | 0/3 | 0/3 | 0/3 | 0/3 | 0/3 |
|  | 14 h | 0/3 | 0/3 | 0/3 | 0/3 | 0/3 |
|  | 24 h | 0/3 | 0/3 | 0/3 | 0/3 | 0/3 |
| 2 | 2 d | 0/3 | 0/3 | 0/3 | 0/3 | 0/3 |
| 3 | 3 d | 0/3 | 0/3 | 0/3 | 0/3 | 0/3 |
| 4 | 4 d | 0/3 | 0/3 | 0/3 | 0/3 | 0/3 |
| 5 | 5 d | 0/3 | 0/3 | 0/3 | 0/3 | 0/3 |
| 6 | 6 d | 0/3 | 0/3 | 0/3 | 0/3 | 0/3 |
| 7 | 7 d | 0/3 | 0/3 | 0/3 | 0/3 | 0/3 |
| 8 | 8 d | 0/3 | 0/3 | 0/3 | 0/3 | 0/3 |
| 9 | 9 d | 0/3 | 0/3 | 0/3 | 0/3 | 0/3 |
| 10 | 10 d | 0/3 | 0/3 | 0/3 | 0/3 | 0/3 |
| 11 | 11 d | 0/3 | 0/3 | 0/3 | 0/3 | 0/3 |
| 12 | 12 d | 0/3 | 0/3 | 0/3 | 0/3 | 0/3 |
| 13 | 13 d | 0/3 | 0/3 | 0/3 | 0/3 | 0/3 |
| 14 | 14 d | 0/3 | 0/3 | 0/3 | 0/3 | 0/3 |

NC = Normal control, VC = 1% DMSO treated normal rats (Vehicle control), T10 = Gαq-RGS2 signaling inhibitor (10 mg/kg) treated rats and T100 = Gαq-RGS2 signaling inhibitor (100 mg/kg) treated rats.

**Table 5.** Effect of single oral dose treatment of Gαq-RGS2 signaling inhibitor on convulsive episode

| **Sr. No.** | **Time** | **Groups**  (Convulsive episode/Total No. of animals) | | | | |
| --- | --- | --- | --- | --- | --- | --- |
|  |  | **NC** | **T1** | **T2** | **T3** | **T4** |
| 1 | 1h | 0/3 | 0/3 | 0/3 | 0/3 | 0/3 |
|  | 2 h | 0/3 | 0/3 | 0/3 | 0/3 | 0/3 |
|  | 4 h | 0/3 | 0/3 | 0/3 | 0/3 | 0/3 |
|  | 14 h | 0/3 | 0/3 | 0/3 | 0/3 | 0/3 |
|  | 24 h | 0/3 | 0/3 | 0/3 | 0/3 | 0/3 |
| 2 | 2 d | 0/3 | 0/3 | 0/3 | 0/3 | 0/3 |
| 3 | 3 d | 0/3 | 0/3 | 0/3 | 0/3 | 0/3 |
| 4 | 4 d | 0/3 | 0/3 | 0/3 | 0/3 | 0/3 |
| 5 | 5 d | 0/3 | 0/3 | 0/3 | 0/3 | 0/3 |
| 6 | 6 d | 0/3 | 0/3 | 0/3 | 0/3 | 0/3 |
| 7 | 7 d | 0/3 | 0/3 | 0/3 | 0/3 | 0/3 |
| 8 | 8 d | 0/3 | 0/3 | 0/3 | 0/3 | 0/3 |
| 9 | 9 d | 0/3 | 0/3 | 0/3 | 0/3 | 0/3 |
| 10 | 10 d | 0/3 | 0/3 | 0/3 | 0/3 | 0/3 |
| 11 | 11 d | 0/3 | 0/3 | 0/3 | 0/3 | 0/3 |
| 12 | 12 d | 0/3 | 0/3 | 0/3 | 0/3 | 0/3 |
| 13 | 13 d | 0/3 | 0/3 | 0/3 | 0/3 | 0/3 |
| 14 | 14 d | 0/3 | 0/3 | 0/3 | 0/3 | 0/3 |

NC = Normal control, VC = 1% DMSO treated normal rats (Vehicle control), T10 = Gαq-RGS2 signaling inhibitor (10 mg/kg) treated rats and T100 = Gαq-RGS2 signaling inhibitor (100 mg/kg) treated rats.

**Table 6.** Effect of single oral dose treatment of Gαq-RGS2 signaling inhibitor on visual place response

| **Sr. No.** | **Time** | **Groups**  (Change in visual place response/Total No. of animals) | | | | |
| --- | --- | --- | --- | --- | --- | --- |
|  |  | **NC** | **T1** | **T2** | **T3** | **T4** |
| 1 | 1h | 0/3 | 0/3 | 0/3 | 0/3 | 0/3 |
|  | 2 h | 0/3 | 0/3 | 0/3 | 0/3 | 0/3 |
|  | 4 h | 0/3 | 0/3 | 0/3 | 0/3 | 0/3 |
|  | 14 h | 0/3 | 0/3 | 0/3 | 0/3 | 0/3 |
|  | 24 h | 0/3 | 0/3 | 0/3 | 0/3 | 0/3 |
| 2 | 2 d | 0/3 | 0/3 | 0/3 | 0/3 | 0/3 |
| 3 | 3 d | 0/3 | 0/3 | 0/3 | 0/3 | 0/3 |
| 4 | 4 d | 0/3 | 0/3 | 0/3 | 0/3 | 0/3 |
| 5 | 5 d | 0/3 | 0/3 | 0/3 | 0/3 | 0/3 |
| 6 | 6 d | 0/3 | 0/3 | 0/3 | 0/3 | 0/3 |
| 7 | 7 d | 0/3 | 0/3 | 0/3 | 0/3 | 0/3 |
| 8 | 8 d | 0/3 | 0/3 | 0/3 | 0/3 | 0/3 |
| 9 | 9 d | 0/3 | 0/3 | 0/3 | 0/3 | 0/3 |
| 10 | 10 d | 0/3 | 0/3 | 0/3 | 0/3 | 0/3 |
| 11 | 11 d | 0/3 | 0/3 | 0/3 | 0/3 | 0/3 |
| 12 | 12 d | 0/3 | 0/3 | 0/3 | 0/3 | 0/3 |
| 13 | 13 d | 0/3 | 0/3 | 0/3 | 0/3 | 0/3 |
| 14 | 14 d | 0/3 | 0/3 | 0/3 | 0/3 | 0/3 |

NC = Normal control, VC = 1% DMSO treated normal rats (Vehicle control), T10 = Gαq-RGS2 signaling inhibitor (10 mg/kg) treated rats and T100 = Gαq-RGS2 signaling inhibitor (100 mg/kg) treated rats.

**Table 7.** Effect of single oral dose treatment of Gαq-RGS2 signaling inhibitor on writhing response

| **Sr. No.** | **Time** | **Groups**  (Writhing response/Total No. of animals) | | | | |
| --- | --- | --- | --- | --- | --- | --- |
|  |  | **NC** | **T1** | **T2** | **T3** | **T4** |
| 1 | 1h | 0/3 | 0/3 | 0/3 | 0/3 | 0/3 |
|  | 2 h | 0/3 | 0/3 | 0/3 | 0/3 | 0/3 |
|  | 4 h | 0/3 | 0/3 | 0/3 | 0/3 | 0/3 |
|  | 14 h | 0/3 | 0/3 | 0/3 | 0/3 | 0/3 |
|  | 24 h | 0/3 | 0/3 | 0/3 | 0/3 | 0/3 |
| 2 | 2 d | 0/3 | 0/3 | 0/3 | 0/3 | 0/3 |
| 3 | 3 d | 0/3 | 0/3 | 0/3 | 0/3 | 0/3 |
| 4 | 4 d | 0/3 | 0/3 | 0/3 | 0/3 | 0/3 |
| 5 | 5 d | 0/3 | 0/3 | 0/3 | 0/3 | 0/3 |
| 6 | 6 d | 0/3 | 0/3 | 0/3 | 0/3 | 0/3 |
| 7 | 7 d | 0/3 | 0/3 | 0/3 | 0/3 | 0/3 |
| 8 | 8 d | 0/3 | 0/3 | 0/3 | 0/3 | 0/3 |
| 9 | 9 d | 0/3 | 0/3 | 0/3 | 0/3 | 0/3 |
| 10 | 10 d | 0/3 | 0/3 | 0/3 | 0/3 | 0/3 |
| 11 | 11 d | 0/3 | 0/3 | 0/3 | 0/3 | 0/3 |
| 12 | 12 d | 0/3 | 0/3 | 0/3 | 0/3 | 0/3 |
| 13 | 13 d | 0/3 | 0/3 | 0/3 | 0/3 | 0/3 |
| 14 | 14 d | 0/3 | 0/3 | 0/3 | 0/3 | 0/3 |

NC = Normal control, VC = 1% DMSO treated normal rats (Vehicle control), T10 = Gαq-RGS2 signaling inhibitor (10 mg/kg) treated rats and T100 = Gαq-RGS2 signaling inhibitor (100 mg/kg) treated rats.

**Table 8.** Effect of single oral dose treatment of Gαq-RGS2 signaling inhibitor on tail pinch response

| **Sr. No.** | **Time** | **Groups**  (Tail pinch response/Total No. of animals) | | | | |
| --- | --- | --- | --- | --- | --- | --- |
|  |  | **NC** | **T1** | **T2** | **T3** | **T4** |
| 1 | 1h | 0/3 | 0/3 | 0/3 | 0/3 | 0/3 |
|  | 2 h | 0/3 | 0/3 | 0/3 | 0/3 | 0/3 |
|  | 4 h | 0/3 | 0/3 | 0/3 | 0/3 | 0/3 |
|  | 14 h | 0/3 | 0/3 | 0/3 | 0/3 | 0/3 |
|  | 24 h | 0/3 | 0/3 | 0/3 | 0/3 | 0/3 |
| 2 | 2 d | 0/3 | 0/3 | 0/3 | 0/3 | 0/3 |
| 3 | 3 d | 0/3 | 0/3 | 0/3 | 0/3 | 0/3 |
| 4 | 4 d | 0/3 | 0/3 | 0/3 | 0/3 | 0/3 |
| 5 | 5 d | 0/3 | 0/3 | 0/3 | 0/3 | 0/3 |
| 6 | 6 d | 0/3 | 0/3 | 0/3 | 0/3 | 0/3 |
| 7 | 7 d | 0/3 | 0/3 | 0/3 | 0/3 | 0/3 |
| 8 | 8 d | 0/3 | 0/3 | 0/3 | 0/3 | 0/3 |
| 9 | 9 d | 0/3 | 0/3 | 0/3 | 0/3 | 0/3 |
| 10 | 10 d | 0/3 | 0/3 | 0/3 | 0/3 | 0/3 |
| 11 | 11 d | 0/3 | 0/3 | 0/3 | 0/3 | 0/3 |
| 12 | 12 d | 0/3 | 0/3 | 0/3 | 0/3 | 0/3 |
| 13 | 13 d | 0/3 | 0/3 | 0/3 | 0/3 | 0/3 |
| 14 | 14 d | 0/3 | 0/3 | 0/3 | 0/3 | 0/3 |

NC = Normal control, VC = 1% DMSO treated normal rats (Vehicle control), T10 = Gαq-RGS2 signaling inhibitor (10 mg/kg) treated rats and T100 = Gαq-RGS2 signaling inhibitor (100 mg/kg) treated rats.

**Table 9.** Effect of single oral dose treatment of Gαq-RGS2 signaling inhibitor on piloerection

| **Sr. No.** | **Time** | **Groups**  (Piloerection/Total No. of animals) | | | | |
| --- | --- | --- | --- | --- | --- | --- |
|  |  | **NC** | **T1** | **T2** | **T3** | **T4** |
| 1 | 1h | 0/3 | 0/3 | 0/3 | 0/3 | 0/3 |
|  | 2 h | 0/3 | 0/3 | 0/3 | 0/3 | 0/3 |
|  | 4 h | 0/3 | 0/3 | 0/3 | 0/3 | 0/3 |
|  | 14 h | 0/3 | 0/3 | 0/3 | 0/3 | 0/3 |
|  | 24 h | 0/3 | 0/3 | 0/3 | 0/3 | 0/3 |
| 2 | 2 d | 0/3 | 0/3 | 0/3 | 0/3 | 0/3 |
| 3 | 3 d | 0/3 | 0/3 | 0/3 | 0/3 | 0/3 |
| 4 | 4 d | 0/3 | 0/3 | 0/3 | 0/3 | 0/3 |
| 5 | 5 d | 0/3 | 0/3 | 0/3 | 0/3 | 0/3 |
| 6 | 6 d | 0/3 | 0/3 | 0/3 | 0/3 | 0/3 |
| 7 | 7 d | 0/3 | 0/3 | 0/3 | 0/3 | 0/3 |
| 8 | 8 d | 0/3 | 0/3 | 0/3 | 0/3 | 0/3 |
| 9 | 9 d | 0/3 | 0/3 | 0/3 | 0/3 | 0/3 |
| 10 | 10 d | 0/3 | 0/3 | 0/3 | 0/3 | 0/3 |
| 11 | 11 d | 0/3 | 0/3 | 0/3 | 0/3 | 0/3 |
| 12 | 12 d | 0/3 | 0/3 | 0/3 | 0/3 | 0/3 |
| 13 | 13 d | 0/3 | 0/3 | 0/3 | 0/3 | 0/3 |
| 14 | 14 d | 0/3 | 0/3 | 0/3 | 0/3 | 0/3 |

NC = Normal control, VC = 1% DMSO treated normal rats (Vehicle control), T10 = Gαq-RGS2 signaling inhibitor (10 mg/kg) treated rats and T100 = Gαq-RGS2 signaling inhibitor (100 mg/kg) treated rats.

**Table 10.** Effect of single oral dose treatment of Gαq-RGS2 signaling inhibitor on lacrimation

| **Sr. No.** | **Time** | **Groups**  (Lacrimation/Total No. of animals) | | | | |
| --- | --- | --- | --- | --- | --- | --- |
|  |  | **NC** | **T1** | **T2** | **T3** | **T4** |
| 1 | 1h | 0/3 | 0/3 | 0/3 | 0/3 | 0/3 |
|  | 2 h | 0/3 | 0/3 | 0/3 | 0/3 | 0/3 |
|  | 4 h | 0/3 | 0/3 | 0/3 | 0/3 | 0/3 |
|  | 14 h | 0/3 | 0/3 | 0/3 | 0/3 | 0/3 |
|  | 24 h | 0/3 | 0/3 | 0/3 | 0/3 | 0/3 |
| 2 | 2 d | 0/3 | 0/3 | 0/3 | 0/3 | 0/3 |
| 3 | 3 d | 0/3 | 0/3 | 0/3 | 0/3 | 0/3 |
| 4 | 4 d | 0/3 | 0/3 | 0/3 | 0/3 | 0/3 |
| 5 | 5 d | 0/3 | 0/3 | 0/3 | 0/3 | 0/3 |
| 6 | 6 d | 0/3 | 0/3 | 0/3 | 0/3 | 0/3 |
| 7 | 7 d | 0/3 | 0/3 | 0/3 | 0/3 | 0/3 |
| 8 | 8 d | 0/3 | 0/3 | 0/3 | 0/3 | 0/3 |
| 9 | 9 d | 0/3 | 0/3 | 0/3 | 0/3 | 0/3 |
| 10 | 10 d | 0/3 | 0/3 | 0/3 | 0/3 | 0/3 |
| 11 | 11 d | 0/3 | 0/3 | 0/3 | 0/3 | 0/3 |
| 12 | 12 d | 0/3 | 0/3 | 0/3 | 0/3 | 0/3 |
| 13 | 13 d | 0/3 | 0/3 | 0/3 | 0/3 | 0/3 |
| 14 | 14 d | 0/3 | 0/3 | 0/3 | 0/3 | 0/3 |

NC = Normal control, VC = 1% DMSO treated normal rats (Vehicle control), T10 = Gαq-RGS2 signaling inhibitor (10 mg/kg) treated rats and T100 = Gαq-RGS2 signaling inhibitor (100 mg/kg) treated rats.

**Table 11.** Effect of single oral dose treatment of Gαq-RGS2 signaling inhibitor on salivation

| **Sr. No.** | **Time** | **Groups**  (Salivation/Total No. of animals) | | | | |
| --- | --- | --- | --- | --- | --- | --- |
|  |  | **NC** | **T1** | **T2** | **T3** | **T4** |
| 1 | 1h | 0/3 | 0/3 | 0/3 | 0/3 | 0/3 |
|  | 2 h | 0/3 | 0/3 | 0/3 | 0/3 | 0/3 |
|  | 4 h | 0/3 | 0/3 | 0/3 | 0/3 | 0/3 |
|  | 14 h | 0/3 | 0/3 | 0/3 | 0/3 | 0/3 |
|  | 24 h | 0/3 | 0/3 | 0/3 | 0/3 | 0/3 |
| 2 | 2 d | 0/3 | 0/3 | 0/3 | 0/3 | 0/3 |
| 3 | 3 d | 0/3 | 0/3 | 0/3 | 0/3 | 0/3 |
| 4 | 4 d | 0/3 | 0/3 | 0/3 | 0/3 | 0/3 |
| 5 | 5 d | 0/3 | 0/3 | 0/3 | 0/3 | 0/3 |
| 6 | 6 d | 0/3 | 0/3 | 0/3 | 0/3 | 0/3 |
| 7 | 7 d | 0/3 | 0/3 | 0/3 | 0/3 | 0/3 |
| 8 | 8 d | 0/3 | 0/3 | 0/3 | 0/3 | 0/3 |
| 9 | 9 d | 0/3 | 0/3 | 0/3 | 0/3 | 0/3 |
| 10 | 10 d | 0/3 | 0/3 | 0/3 | 0/3 | 0/3 |
| 11 | 11 d | 0/3 | 0/3 | 0/3 | 0/3 | 0/3 |
| 12 | 12 d | 0/3 | 0/3 | 0/3 | 0/3 | 0/3 |
| 13 | 13 d | 0/3 | 0/3 | 0/3 | 0/3 | 0/3 |
| 14 | 14 d | 0/3 | 0/3 | 0/3 | 0/3 | 0/3 |

NC = Normal control, VC = 1% DMSO treated normal rats (Vehicle control), T10 = Gαq-RGS2 signaling inhibitor (10 mg/kg) treated rats and T100 = Gαq-RGS2 signaling inhibitor (100 mg/kg) treated rats.

**Table 12.** Effect of single oral dose treatment of Gαq-RGS2 signaling inhibitor on sniffing

| **Sr. No.** | **Time** | **Groups**  (Sniffing/Total No. of animals) | | | | |
| --- | --- | --- | --- | --- | --- | --- |
|  |  | **NC** | **T1** | **T2** | **T3** | **T4** |
| 1 | 1h | 0/3 | 0/3 | 0/3 | 0/3 | 0/3 |
|  | 2 h | 0/3 | 0/3 | 0/3 | 0/3 | 0/3 |
|  | 4 h | 0/3 | 0/3 | 0/3 | 0/3 | 0/3 |
|  | 14 h | 0/3 | 0/3 | 0/3 | 0/3 | 0/3 |
|  | 24 h | 0/3 | 0/3 | 0/3 | 0/3 | 0/3 |
| 2 | 2 d | 0/3 | 0/3 | 0/3 | 0/3 | 0/3 |
| 3 | 3 d | 0/3 | 0/3 | 0/3 | 0/3 | 0/3 |
| 4 | 4 d | 0/3 | 0/3 | 0/3 | 0/3 | 0/3 |
| 5 | 5 d | 0/3 | 0/3 | 0/3 | 0/3 | 0/3 |
| 6 | 6 d | 0/3 | 0/3 | 0/3 | 0/3 | 0/3 |
| 7 | 7 d | 0/3 | 0/3 | 0/3 | 0/3 | 0/3 |
| 8 | 8 d | 0/3 | 0/3 | 0/3 | 0/3 | 0/3 |
| 9 | 9 d | 0/3 | 0/3 | 0/3 | 0/3 | 0/3 |
| 10 | 10 d | 0/3 | 0/3 | 0/3 | 0/3 | 0/3 |
| 11 | 11 d | 0/3 | 0/3 | 0/3 | 0/3 | 0/3 |
| 12 | 12 d | 0/3 | 0/3 | 0/3 | 0/3 | 0/3 |
| 13 | 13 d | 0/3 | 0/3 | 0/3 | 0/3 | 0/3 |
| 14 | 14 d | 0/3 | 0/3 | 0/3 | 0/3 | 0/3 |

NC = Normal control, VC = 1% DMSO treated normal rats (Vehicle control), T10 = Gαq-RGS2 signaling inhibitor (10 mg/kg) treated rats and T100 = Gαq-RGS2 signaling inhibitor (100 mg/kg) treated rats.

**Table 13.** Effect of single oral dose treatment of Gαq-RGS2 signaling inhibitor on defecation

| **Sr. No.** | **Time** | **Groups**  (Defecation/Total No. of animals) | | | | |
| --- | --- | --- | --- | --- | --- | --- |
|  |  | **NC** | **T1** | **T2** | **T3** | **T4** |
| 1 | 1h | 0/3 | 0/3 | 0/3 | 0/3 | 0/3 |
|  | 2 h | 0/3 | 0/3 | 0/3 | 0/3 | 0/3 |
|  | 4 h | 0/3 | 0/3 | 0/3 | 0/3 | 0/3 |
|  | 14 h | 0/3 | 0/3 | 0/3 | 0/3 | 0/3 |
|  | 24 h | 0/3 | 0/3 | 0/3 | 0/3 | 0/3 |
| 2 | 2 d | 0/3 | 0/3 | 0/3 | 0/3 | 0/3 |
| 3 | 3 d | 0/3 | 0/3 | 0/3 | 0/3 | 0/3 |
| 4 | 4 d | 0/3 | 0/3 | 0/3 | 0/3 | 0/3 |
| 5 | 5 d | 0/3 | 0/3 | 0/3 | 0/3 | 0/3 |
| 6 | 6 d | 0/3 | 0/3 | 0/3 | 0/3 | 0/3 |
| 7 | 7 d | 0/3 | 0/3 | 0/3 | 0/3 | 0/3 |
| 8 | 8 d | 0/3 | 0/3 | 0/3 | 0/3 | 0/3 |
| 9 | 9 d | 0/3 | 0/3 | 0/3 | 0/3 | 0/3 |
| 10 | 10 d | 0/3 | 0/3 | 0/3 | 0/3 | 0/3 |
| 11 | 11 d | 0/3 | 0/3 | 0/3 | 0/3 | 0/3 |
| 12 | 12 d | 0/3 | 0/3 | 0/3 | 0/3 | 0/3 |
| 13 | 13 d | 0/3 | 0/3 | 0/3 | 0/3 | 0/3 |
| 14 | 14 d | 0/3 | 0/3 | 0/3 | 0/3 | 0/3 |

NC = Normal control, VC = 1% DMSO treated normal rats (Vehicle control), T10 = Gαq-RGS2 signaling inhibitor (10 mg/kg) treated rats and T100 = Gαq-RGS2 signaling inhibitor (100 mg/kg) treated rats.
